# Hybrid Response Surface–Particle Swarm Optimisation of Donnan Dialysis Processes for Aluminium Recovery from Water Treatment Sludge

**DOI:** 10.3390/membranes16080256

**Published:** 2026-07-27

**Authors:** James Darmey, Sudesh Rathilal, Emmanuel Kweinor Tetteh, Julius Cudjoe Ahiekpor

**Affiliations:** 1 Green Engineering Research Group, Department of Chemical Engineering, Faculty of Engineering and the Built Environment, Durban University of Technology, Steve Biko, S4 l1, Durban 4001, South Africa; 2 Faculty of Engineering, the Built Environment and Technology, Nelson Mandela University, Gardham Avenue, Gqeberha 6031, South Africa; 3 Chemical Engineering Department, Kumasi Technical University, Kumasi P.O. Box 854, Ghana

**Keywords:** coagulant recovery, optimisation, Donnan dialysis, waste management, water treatment plant sludge

## Abstract

Sustainable recovery of aluminium from water treatment plant sludge (WTPS) offers a promising route for resource valorisation and waste reduction. In this study, Donnan dialysis (DD) is evaluated as a separation technique for recovering aluminium from a synthetic hydroxide-based feed simulating WTPS. A Box–Behnken design coupled with response surface methodology (BBD–RSM) was employed to model and quantify the effects of key operating parameters, namely feed pH, flow rate, initial aluminium concentration, runtime, and sweep solution concentration. Particle Swarm Optimisation (PSO) was integrated with the RSM framework to enhance global optimisation. The PSO approach predicted a maximum aluminium recovery of 99.1% under optimal conditions (pH 4.74, flow rate 98.60%, feed concentration 1313.3 ppm, runtime 21.5 h, and sweep concentration 0.25 M). Experimental validation yielded a recovery efficiency of 90.4%, corresponding to a deviation of 9.2% at a 95% confidence level with R^2^ = 0.9632 and predicted R^2^ = 0.9072. The results demonstrate that DD is an effective and scalable approach for recovering aluminium from hydroxide-rich sludge matrices, while PSO provides a robust optimisation strategy to address the nonlinearities inherent in membrane-based separation processes. This hybrid modelling framework advances process optimisation methodologies and supports the development of sustainable sludge-to-resource technologies in water treatment systems.

## 1. Introduction

Aluminium (Al) salts, particularly aluminium sulphate (alum), are extensively employed as coagulants in drinking water and wastewater treatment worldwide, playing a crucial role in removing colloidal particles, turbidity, and natural organic matter [1]. Globally, water treatment plants generate vast amounts of aluminium-rich sludge, with estimates indicating that over 100 million tons are produced annually, posing significant environmental challenges [2]. In Africa, where access to safe drinking water remains a concern for over 40% of the population [3], the management of water treatment byproducts is even more critical. The disposal of this sludge often results in environmental contamination when it is discharged into rivers and lakes or sent to landfills, leading to the leaching of aluminium into water bodies. Elevated aluminium levels pose toxicity risks to wildlife, aquatic organisms, and humans, causing neurological and developmental issues [4,5]. Addressing these concerns by recovering aluminium from water treatment sludge (WTPS) offers an innovative, sustainable approach that can mitigate environmental impact while generating valuable resources [2,6].

Conventionally, techniques such as acid digestion, alkalization, ion exchange, and membrane-based technologies have been developed for aluminium extraction. Among membrane-based technologies, Donnan dialysis (DD) stands out for its simplicity, energy efficiency, and effectiveness. It employs a cation-exchange membrane that permits positively charged ions such as aluminium to pass through while blocking negatively charged and uncharged species. This process relies on an electrochemical potential gradient [7,8,9], operates without transmembrane pressure, and avoids pore fouling, a common issue in reverse osmosis, ultrafiltration, and nanofiltration technologies [9]. Two modes govern the DD Al ions transport process. The first equation, the Nernst–Planck equation (Equation (1)), describes the amount of permeate generated per unit area of the membrane surface per unit time [10]. This equation is used to determine ion movement across the DD membrane. The second critical equation (Equation (2)) that clarifies the relationship between initial and final concentrations of the solutions used in the feed and sweep phases is proposed by Davis [2].

(1) $$J_{m}=-D_{m}\left(\frac{dC_{m}}{dt}+Z_{i}C_{m}\frac{F}{RT}\frac{d\emptyset }{dl}\right)$$

where $D_{m}$ presents the diffusivity (m^2^ s^−1^), Cm reflects the metal ion bulk solution concentration (mol m^−3^), and Jm indicates the flux. The membrane thickness is represented as (m), T refers to revised absolute temperature (K), Z represents the ionic charge, F denotes Faraday’s constant (C mol^−1^), ∅ is defined as the electric potential, and R is the ideal constant (J K^−1^ mol^−1^).

(2) $${\left(\frac{{\left[C_{a}\right]}_{f^{+}}}{{\left[C_{a}\right]}_{s^{+}}}\right)}^{\frac{1}{Z_{a}}}={\left(\frac{{\left[C_{b}\right]}_{f^{+}}}{{\left[C_{b}\right]}_{s^{+}}}\right)}^{\frac{1}{Z_{b}}}$$

The ions in the solution of the feed phase are represented by subscripts f and s. The concentration of the target metal is designated as $C_{a}$, the ionic charge as Za, and the concentration and ionic charge of the ionic species replacing the target metal are designated as $C_{b},$ $Z_{b}$, respectively. Therefore, a high value of $C_{b}$ will increase the amount of target metal, $C_{a}$, that is ultimately recovered, and the recovery and concentration of the target metal ion will be even higher if the target metal ion has a larger $Z_{a}$ than $Z_{b}$ (the ionic species that it is substituting).

Implementing such innovative recovery methods is particularly significant in Africa, where sustainable water management solutions are urgently needed to improve water quality, reduce waste, and promote environmental health.

Prakash and SenGupta [8] demonstrated that utilizing a Nafion 117 homogeneous cation-exchange membrane in Donnan dialysis (DD) enables the recovery of over 70% of aluminium from water treatment residuals (WTRs) in a single-step process. Building upon these findings, Prakash et al. [3] further compared the performance of Nafion 117 with the heterogeneous Ionac 3470 membrane specifically for aluminium recovery. Their results indicated that Nafion 117 achieved an impressive recovery rate of approximately 80% [7]. Despite these promising results, it is important to note that the efficiency of DD depends heavily on several critical factors, including feed and sweeping concentrations, pH levels, membrane characteristics, and flow rates. DD has some limitations, mainly because many DD studies focus on a limited set of key parameters like feed concentration, sweep concentration, and flow rate. However, other critical factors such as pH, duration, temperature, the type and composition of the sweep solution, and the valence and hydrated radius of ions also play essential roles in ion separation [11,12].

To optimise Donnan dialysis (DD), it is crucial to thoroughly understand the variables that influence it. The application of Response Surface Methodology (RSM) has been explored, particularly to improve aluminium recovery and enable reliable predictions of outcomes from water treatment sludge [5,13]. As described by Bezerra et al. [14], RSM is a versatile and widely used analytical technique that integrates statistical and mathematical tools to develop, model, analyse, and optimise processes involving multiple interdependent variables [5,15]. Furthermore, combining RSM with the Box–Behnken design (BBD) enhances the optimisation of DD parameters and provides insight into the relationships between input factors and alum recovery by analysing three factorial points [7]. Pillay et al. [9] demonstrated the effectiveness of this combined approach in optimising DD using a Nafion 117 membrane, showing that the quadratic statistical model was statistically significant (*p* < 0.001) and accurately predicted alum recoveries between 85% and 96% [7]. Therefore, modelling and optimising DD with RSM offer a systematic framework that not only improves process efficiency but also minimises experimental effort. This approach is essential for scaling up industrial applications, ensuring water treatment processes are both effective and economically viable. It primarily relies on low-order polynomial models, such as second-order polynomials, which, while generally adequate, may not fully capture the highly nonlinear behaviours observed in complementary tools, such as combining RSM with machine learning algorithms, such as Particle Swarm Optimisation (PSO), artificial neural networks (ANNs) and support vector machines (SVMs), which can enhance its capabilities by capturing highly nonlinear relationships and managing large datasets [13,16].

Furthermore, another limitation in DD Al recovery studies is the choice of feed solution. Aluminium hydroxide is the primary aluminium compound in WTPS [17], not Al_2_(SO_4_)_3_, which has been used in the feed solution [18]. Aluminium sulphate dissolves readily in water, providing Al^3+^ ions necessary for transport across the ion-exchange membrane. In contrast, aluminium hydroxide has limited solubility and tends to form precipitates, which can alter the kinetics and models of the DD Al recovery process. This leads to complex, nonlinear interactions among operating variables that can restrict the effectiveness of traditional optimisation techniques. A Box–Behnken design within response surface methodology (BBD–RSM) was employed to evaluate the effects and interactions of pH, runtime, feed concentration, sweep concentration, and feed flow rate. Although hybrid RSM–Particle Swarm Optimisation (PSO) methods are common in chemical and separation processes, their use in Donnan dialysis for recovering aluminium from hydroxide-rich WTPS has not been documented. This study introduces PSO with an RSM model framework to optimise Donnan dialysis under realistic sludge conditions, assess the drawbacks of traditional RSM optimisation, and define experimentally validated operating conditions to improve aluminium recovery. The key innovation is the application of BBD-PSO to Donnan dialysis for aluminium extraction from a hydroxide-rich feed typical of WTPS.

## 2. Methodology

### 2.1. Design of Experiment

In this study, a methodical experimental design was developed using Design-Expert (13) software (Stat-Ease, Inc., Minneapolis, MN, USA) to collect sufficient data to optimise the procedure with as few experiments as possible. The goal was to determine the optimal range of operational parameters for controlling, optimising, and improving the effectiveness of the DD coagulant recovery process. A BBD with 43 experiments and 5 input factors was conducted under random conditions. The percentage recovery of Al was the response, and the response surface methodology–Box–Behnken design (RSM-BBD) was used to evaluate interactions among the factors of concern at three levels: low (−1), medium (0), and high (+1). The 43 experimental runs, including 40 factorial edge points and three replicated centre points, were used to estimate pure experimental error and evaluate model adequacy. To reduce systematic bias from uncontrolled variables, the runs were conducted in a randomized order generated by the Design-Expert software. The low and high extremes were 1–5 for feed pH (X_1_), 20–100% of 3.2 mL/s for feed flow rate (X_2_), 50–4000 mg/L of Al(OH)_3_ solution for feed concentration (X_3_), 0.5–24 h for runtime (X_4_), and 0.25–1 M for sweep concentration (X_5_), as shown in Table 1. These parameters were found to affect Al recovery using DD, except for sweep flow rate, which was kept constant in this study [7].

The percentage recovery of Al^3+^ from the feed solution was calculated using Equation (3):

(3) $$\mathrm{Al}\%=\frac{C_{o}-C_{f}}{C_{o}}\times 100\%$$

where C_o_ is the initial Al concentration of the feed solution, and C_f_ is the final Al concentration of the feed solution after the DD recovery process. A Nafion 117 commercial cation-exchange membrane manufactured by DuPont Inc. was used in this study. The DD Al recovery process method by Asante-Sackey et al. [19] was adapted in this study.

### 2.2. Donnan Dialysis Al Recovery Setup

**Figure 1:**
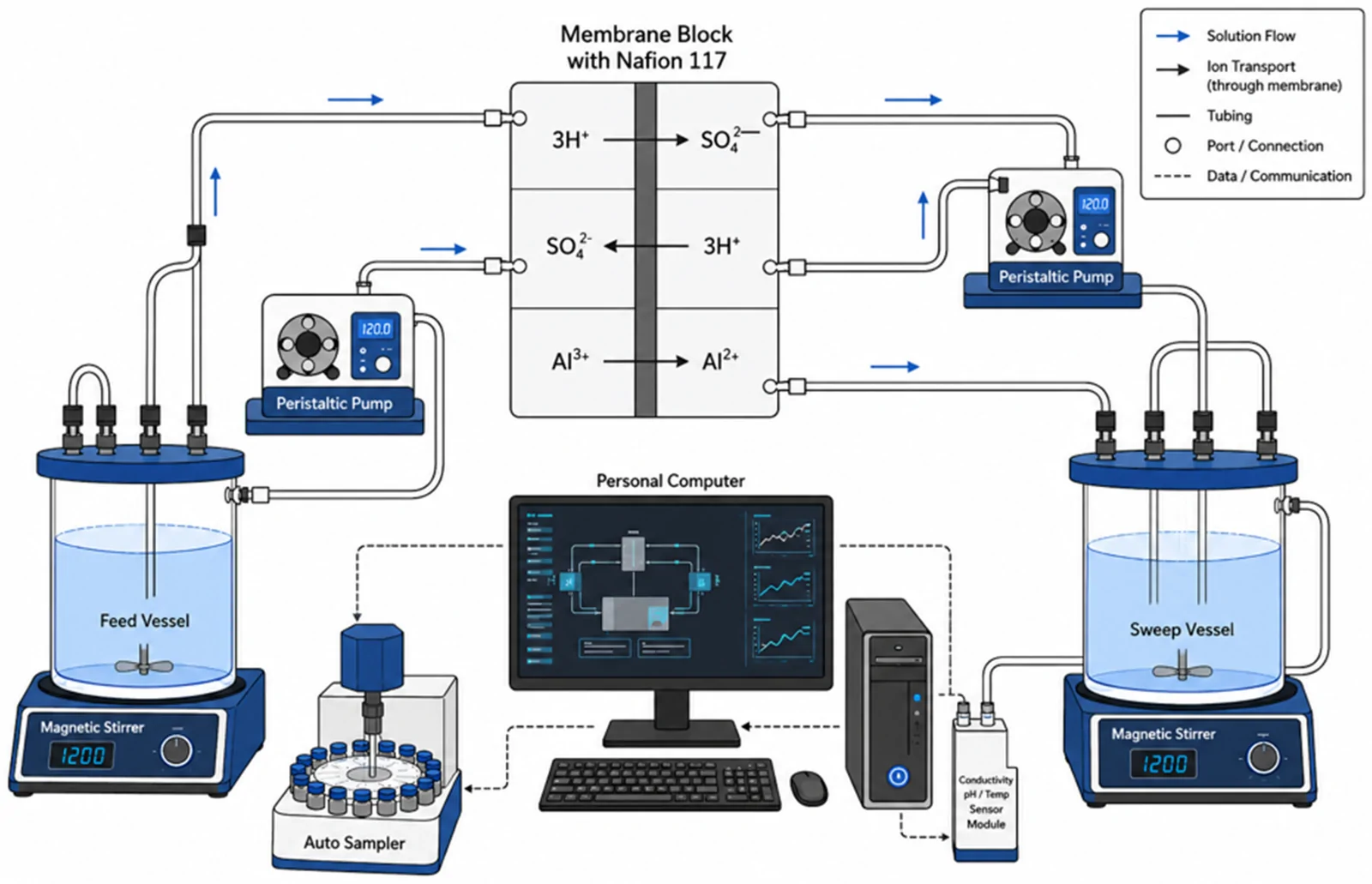
Schematic diagram of *Donnan dialysis Al recovery experimental setup in operation*.

This experiment utilised a flat sheet membrane module. PVC makes up the rig block that houses the membrane. The overall volume of each block is 2114 cm^3^. The equivalent weight, thickness, working area and ion exchange capacity of the Nafion membrane used are 1100 g mol^−1^, 177.8 µm, 205 cm^2^ and 0.94 meq/g, respectively. Using a 3 mm thick, acid-resistant rubber gasket to seal the block on both sides of the membrane prevents leaks. The PTFE tubes with an internal ID of 12 mm are used to connect the membrane rig to the peristaltic pumps of the feed and sweep vessels, each with a working volume of 2 litres. Figure 1 shows the experimental setup. In this study, synthetic WTPS feeds were made from Al(OH)_3_ and used as feed solutions, while H_2_SO_4_ solutions served as sweep solutions. Synthetic WTPS was prepared by dispersing analytical-grade Al(OH)_3_ in deionised water to mimic the aluminium hydroxide-rich composition of real WTPS. The suspension pH was subsequently adjusted with dilute H_2_SO_4_ to the desired experimental pH (1–5) to produce acidified synthetic WTPS. Acidification partially dissolved the aluminium hydroxide, generating soluble aluminium species available for Donnan dialysis. The membrane was treated, encased in the PVC block, and fastened together. Using the experimental matrices displayed in Table S1, a variable flow rate peristaltic pump (A1N31F-7T) with a maximum flow rate of 3.2 mL/s (varied from 20–100%) was used to pump the acidified synthetic WPTS from the feed tank to the Nafion 117 cation exchange membrane, and the peristaltic pump (A1N31F-7T) with a maximum flow rate of 2.6 mL/s (at a 100% opening) was used to circulate the sweep solution. The recirculating counterflow process between the feed solution and the specified sweep solution was conducted for durations of 0.5 to 24 h. As shown in the actual setup for the DD process (Figure 1), the feed vessel on the left of the membrane block, the sweep vessel on the right side of the membrane block, and magnetic stirrers were used to agitate the electrolytic solutions in the feed and sweep vessels. Samples were collected before and after the setup’s operation from the feed and sweep tanks for each experimental run. After each run, the pumps are allowed to run to drain all accumulated solution in the tubes in the vessels. The analysis of dissolved Al ions in the filtered acidified synthetic WTPS and final sweep solutions was carried out using an atomic absorption spectrometer (AAS Vario6, Analytical Jena GmbH, Jena, Germany). At the end of each run, the feed and sweep tanks were then filled with deionized water. The pumps were operated at mid-flow rates to circulate the deionized water to clean the rig. The recirculation and cleaning process was completed an hour before the pumps were switched off and tanks emptied. The Nafion membrane was then removed from the PVC housing and saturated in 1 wt. % HCl and rinsed for 15 min in demineralized water before it was replaced back on the block for another run.

### 2.3. Modelling of DD Al Recovery Process

In this study, RSM-BBD was executed to examine the effects of 5 independent variables: pH of the feed solution (X_1_), feed flow rate (X_2_), feed concentration (X_3_), runtime (X_4_), and sweep concentration (X_5_), on the DD recovery efficiency. An experimental DD recovery design was developed using a BBD implemented in Design-Expert software (version 13.0.5.0). All experiments were carried out at room temperature, with a sweep flow rate of 2.7 mL/s. A five-factor and level (–α, 1, 0, 1, and α) study consisting of 43 experiments was designed to model and optimise the DD coagulant recovery process. The 43 experimental runs are summarised in Table 2. The experimental matrix was followed, and the response data were generated. The response data were entered and statistically analysed. The response models were verified by comparing their predicted values with the experimental results. The values of the regression parameters, that is, the correlation coefficient R^2^, adjusted R^2^, F-value, and *p*-value, were calculated using variance analysis (ANOVA) and used to determine the relevance and suitability of the predicted model. Based on the 95% confidence level in the developed model, the significance of the independent variables on the DD coagulant recovery process was evaluated. Equation (4) was used to describe the impact of variables in terms of linear, quadratic and cross-product terms [20].

(4) $$Y\%=a_{0}+\sum_{i=1}^{k}\beta_{i}X_{i\;}+\sum_{i=1}^{k}\beta_{ij}X_{i}^{2}+\sum_{i<j}^{k}\sum_{j}^{k}\beta_{ij}X_{i}X_{j}+e$$

where Y refers to Al recovery or the change in conductivity of the feed solution, while k is the number of factors studied in the experiment. β and ‘*e*’ are the regression coefficients and the residual error, respectively.

### 2.4. Optimisation of DD Coagulant Recovery Process

BBD-RSM in Design-Expert 13 (Stat-Ease, Inc., USA) and PSO in Python 3.13 (by Python Software Foundation, Minneapolis, MN, USA) were used to optimise the DD Al recovery process. PSO was used to optimise Al recovery using the PySwarms library in Python. In this study, optimisation was performed without adjustment of factor values. That is, all factors are within their corresponding ranges, while the response is set to its maximum value. After, the optimised values were validated experimentally. All validation experiments were performed in triplicate, and the reported value represents the mean.

## 3. Results and Discussions

### 3.1. Design of Experiment and Statistical Analysis

Aluminium speciation is highly dependent on pH. At pH 1–5, aluminium exists predominantly as dissolved monomeric hydrolysis species such as Al^3+^, AlOH^2+^ and Al(OH)_2_^+^, while precipitation of amorphous Al(OH)_3_ becomes increasingly important above a pH of 5. Since all experiments were conducted at a pH of 5 and below, soluble cationic species dominated and constituted the mobile ions transported across the Nafion 117 membrane.

The experimental runs for the main and interaction effects of input variables were designed using the BBD, as shown in Table S1. A statistical model was developed using RSM. To validate the reliability of the experimental transport data and monitor accumulation and aluminium loss or precipitation, a mass balance and aluminium loss analysis was conducted across all 43 runs. The mass balance demonstrated continuity, as shown in Table S2, with unaccounted-for dissolved aluminium remaining at 0.0% across 97.6% of the experimental matrix. This confirms that the reduction of dissolved aluminium ions in the feed phase corresponds directly to clean membrane transport into the acid sweep phase.

As mentioned earlier, 43 runs were conducted using a Box–Behnken design to investigate the effects of five variables on Al recovery (%). The statistical parameters were estimated by using ANOVA (Table 2 and Table 3). According to the Design-Expert output, the two-factor interaction (2FI) model was not aliased. This happens when the effect of one input parameter depends on the level of another. The final empirical model, expressed as a coded factor for Al recovery (Y, %), is given in Equation (5), and the actual factors are given in Equation (6). Feed pH, feed concentration, feed flow rate, runtime, and sweep concentration were evaluated for their degree of influence and significance using the Fisher test (F-test; F-value) and the probability value (*p*-value; α = 0.05). A *p*-value < α indicates a statistically significant link between the factors and the response (Al recovery). A statistically significant relationship between the response (Al recovery) and the factors is denoted by *p*-value ≤ α. The total variability of the data is attributed to either specific causes or to the model’s simulation. The data and ANOVA analysis are shown in Table 2.

(5) $$Y\%=45.04+8A-1.69B-8.06C+6.19D+4.44E+4.75\left(AB\right)+22.50\left(AC\right)\;+18.25\left(AD\right)-4.50\left(AE\right)-5.50\left(BC\right)-4.00\left(BD\right)-20.00\left(BE\right)+13.00\left(CD\right)\;+14.25\left(CE\right)-23.00(DE)$$

(6) $$Y\%=26.14539-16.86061\left(A\right)+0.858257\left(B\right)-0.035881\left(C\right)+0.8354565\left(D\right)\;+134.81457\left(E\right)+0.059375\left(AB\right)+0.0055696\left(AC\right)+0.776596\left(AD\right)\;-6.0000\left(AE\right)-0.000070\left(BC\right)-0.008511\left(BD\right)-1.33333\left(BE\right)\;+0.000560\left(CD\right)+0.019241\left(CE\right)-5.21986(DE)$$

Y% = Percentage Al recovery
A = pH of feed solution
B = Flow rate of feed solution,
C = Feed concentration, ppm
D = Runtime, h
E = Sweep concentration, M

In the models, a positive sign indicates a synergistic effect, whereas a negative sign indicates an antagonistic effect [21]. The greatest negative impact from an independent term in Equation (3) was feed concentration. This confirms that increasing the feed concentration reduces Al recovery, as suggested by Asante-Sackey et al. [7]. The greatest positive effects arise from the combined action of runtime (D) and sweep concentration (E), suggesting that to maximise Al recovery, D and E should be set at similar levels. The model’s predictive accuracy is indicated by the coefficient of determination (R^2^), which is 0.5503 and far from one. This means Equation (5) has moderate explanatory power in the Al recovery DD process. From Table 3, a negative predicted R^2^ implies that the overall mean may be a better predictor of your response than the current model. In some cases, a higher-order model may also provide better predictions. Adequate precision measures the signal-to-noise ratio. A ratio greater than four is desirable. A ratio of 6.1192 indicates an adequate signal. This model can be used to navigate the design space. The lack of fit F-value of 2344.34 implies that the “lack of fit” is significant. The larger *p*-values (for “lack of fit” > 0.05) in Table 3 indicate that the F statistic is insignificant. There is only a 0.01% chance that a “lack of fit F-value” occurs due to noise. This means the model must be optimised, as it does not adequately describe the relationship between the input parameters and the response.

### 3.2. ANOVA of Reduced Cubic DD Al Recovery Model

To further optimise the model, some cubic input terms were included in the 2FI model, and the predicted recoveries were included in the modelling, to obtain a reduced cubic model in Equations (7) (coded equation) and (8) (actual equation). The reduced cubic model was selected after comparing several candidate polynomial models using sequential model sum-of-squares, lack-of-fit analysis, adjusted and predicted R^2^ values, adequate precision, and residual diagnostics. When comparing the *p*-values and F-values of the reduced cubic model in Table 4 and the 2FI model in Table 2, the reduced cubic model is the most significant, with less than 0.05% chance that an F-value would occur by chance. Additionally, the reduced cubic model has higher adequate precision, as shown in Table 5, and will therefore be more desirable for navigating the design space. The predicted R^2^ of 0.9072 is in reasonable agreement with the adjusted R^2^ of 0.9485; that is, the difference is less than 0.2. This is better than what was obtained for the unoptimized 2FI model. This means the reduced cubic model has better predictive ability than the 2FI model, proving why the cubic model was selected. The R^2^ value of 0.9632 for the reduced cubic DD Al recovery process model is optimal, as shown in Table 4, and exceeds that of the 2FI model. This means the reduced cubic model is a good fit for the DD Al recovery process. Although the increase in R^2^ relative to the initial 2FI model appears substantial, the close agreement between the adjusted R^2^ (0.9485) and predicted R^2^ (0.9072) indicates that the model retained good predictive capability and was not excessively overfitted. Adequate precision of 34.28 indicates an adequate signal better than that of the 2FI model.

(7) $$Y\%=44.26+8A-1.69B-8.06C+6.19D+0.7925E+22.50\left(AC\right)+18.25\left(AD\right)\;-20.00\left(BE\right)+14.25\left(CE\right)-23.00\left(DE\right)+7.29\left(B^{2}E\right)+7.29\left(C^{2}E\right)$$

(8) $$Y\%=26.4999-17.04811A+0.79115B-0.033196C+1.45911D+121.27005E\;+0.005696\left(AC\right)+0.776596\left(AD\right)-145253\left(BE\right)+0.01759\left(CE\right)\;-5.21986\left(DE\right)-0.00099\left(B^{2}E\right)+4.07455\times 10^{-7}\left(C^{2}E\right)$$

where

Y% = Percentage Al recovery
A = pH of feed solution
B = Flow rate of feed solution,
C = Feed concentration, ppm
D = Runtime, h
E = Sweep concentration, M

The highest negative impact from an independent term from Equation (7) was the feed concentration, which agrees with that obtained in Equation (5). The greatest positive effects arise from the combined action of the square of feed pH (A) and feed concentration (C), suggesting that to maximise Al recovery, A and C should be set at similar levels.

### 3.3. Model Evaluation Plots of the Reduced Cubic DD Al Recovery Model

**Figure 2:**
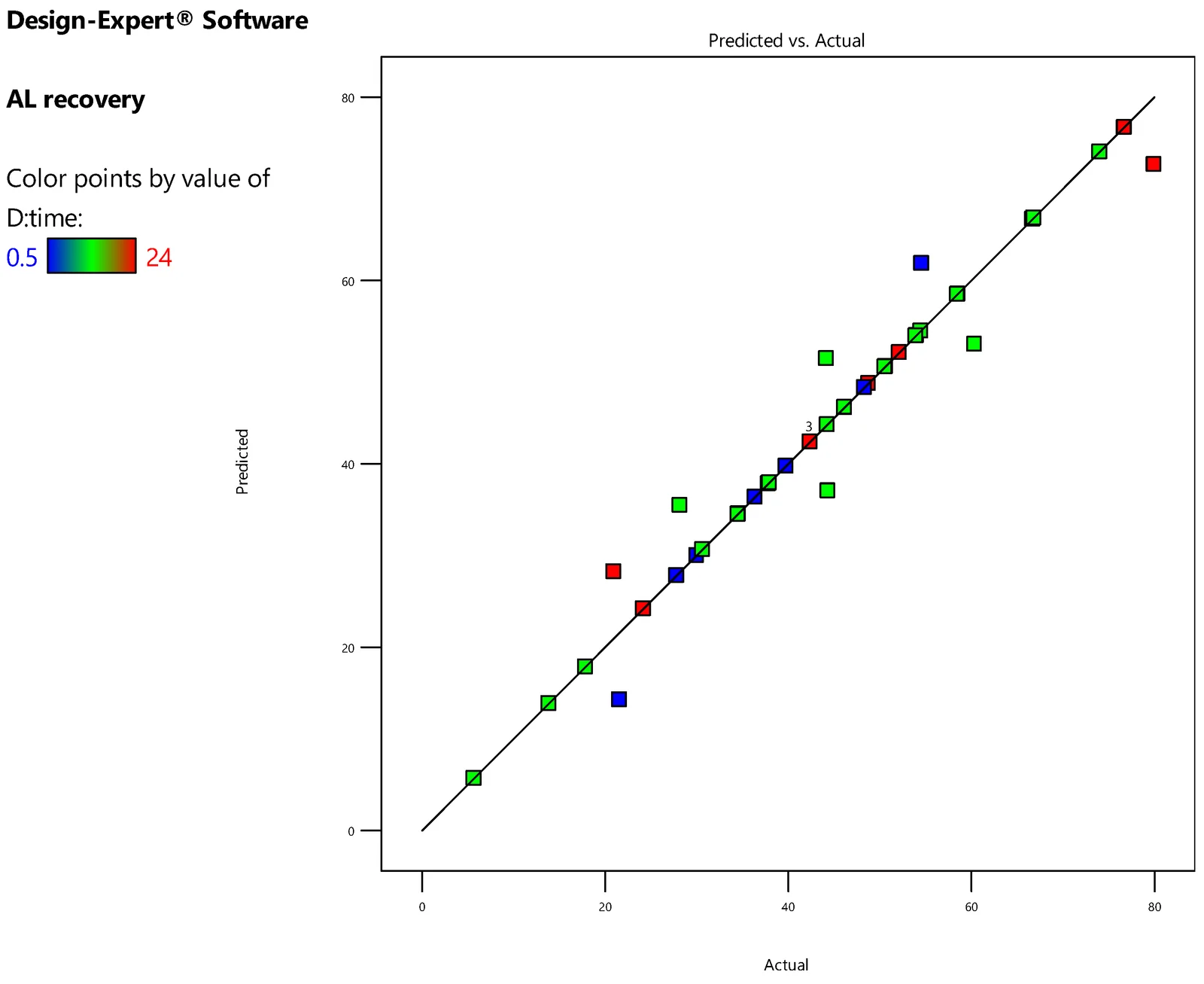
Predicted versus actual plot of DD Al recovery.

The predicted-versus-actual plot in Figure 2 shows points close to the diagonal line for aluminium recovery. This indicates a strong correlation between the predictions of the reduced cubic DD Al recovery model and the actual experimental data. The alignment of the points indicates that the models correspond effectively with the experimental data.

**Figure 3:**
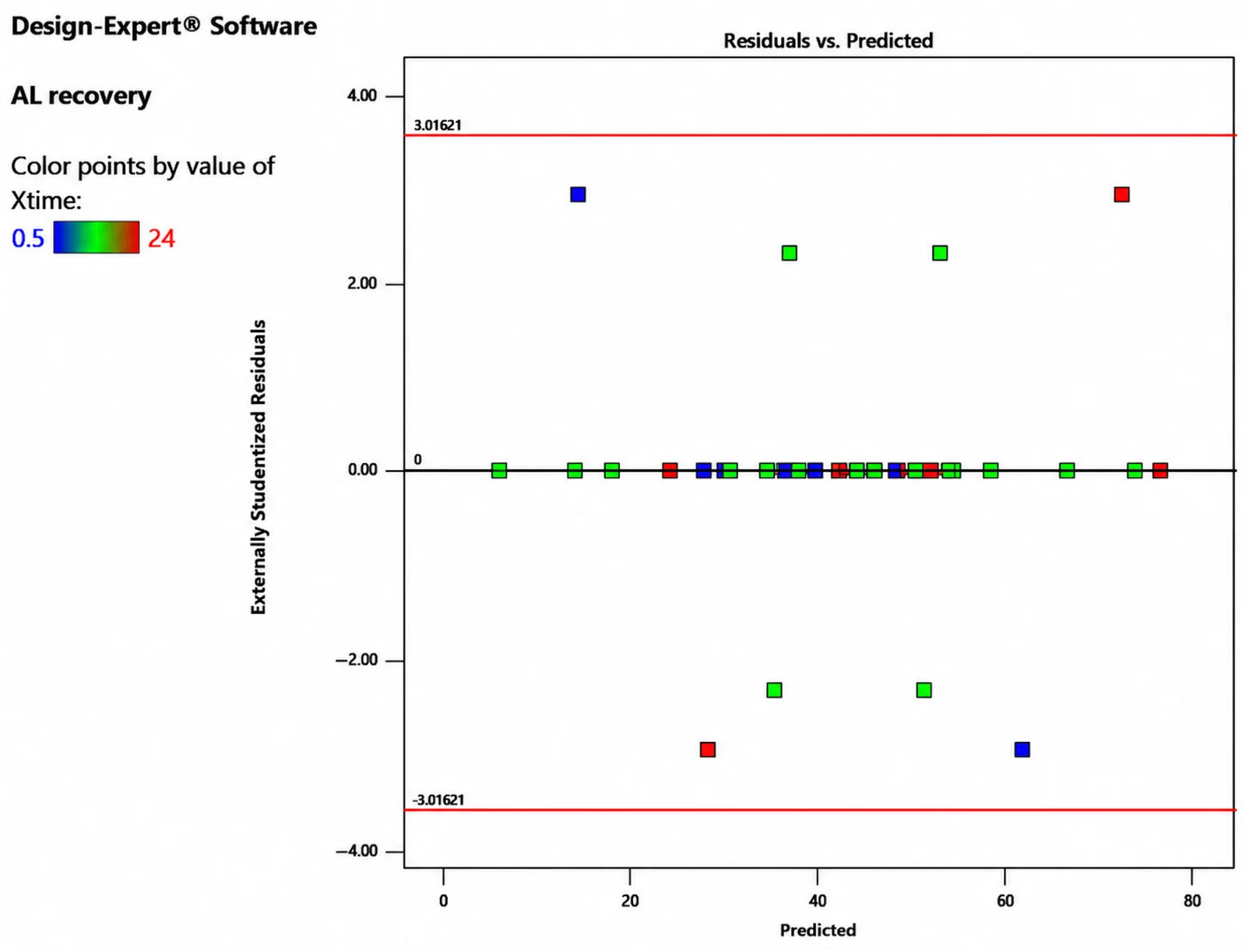
The residuals versus predicted values plots for the reduced cubic model.

Figure 3 shows the plot of residuals against the expected values for the models. The points remain consistently within the graph’s range, indicating that the models accurately represent the experimental data.

**Figure 4:**
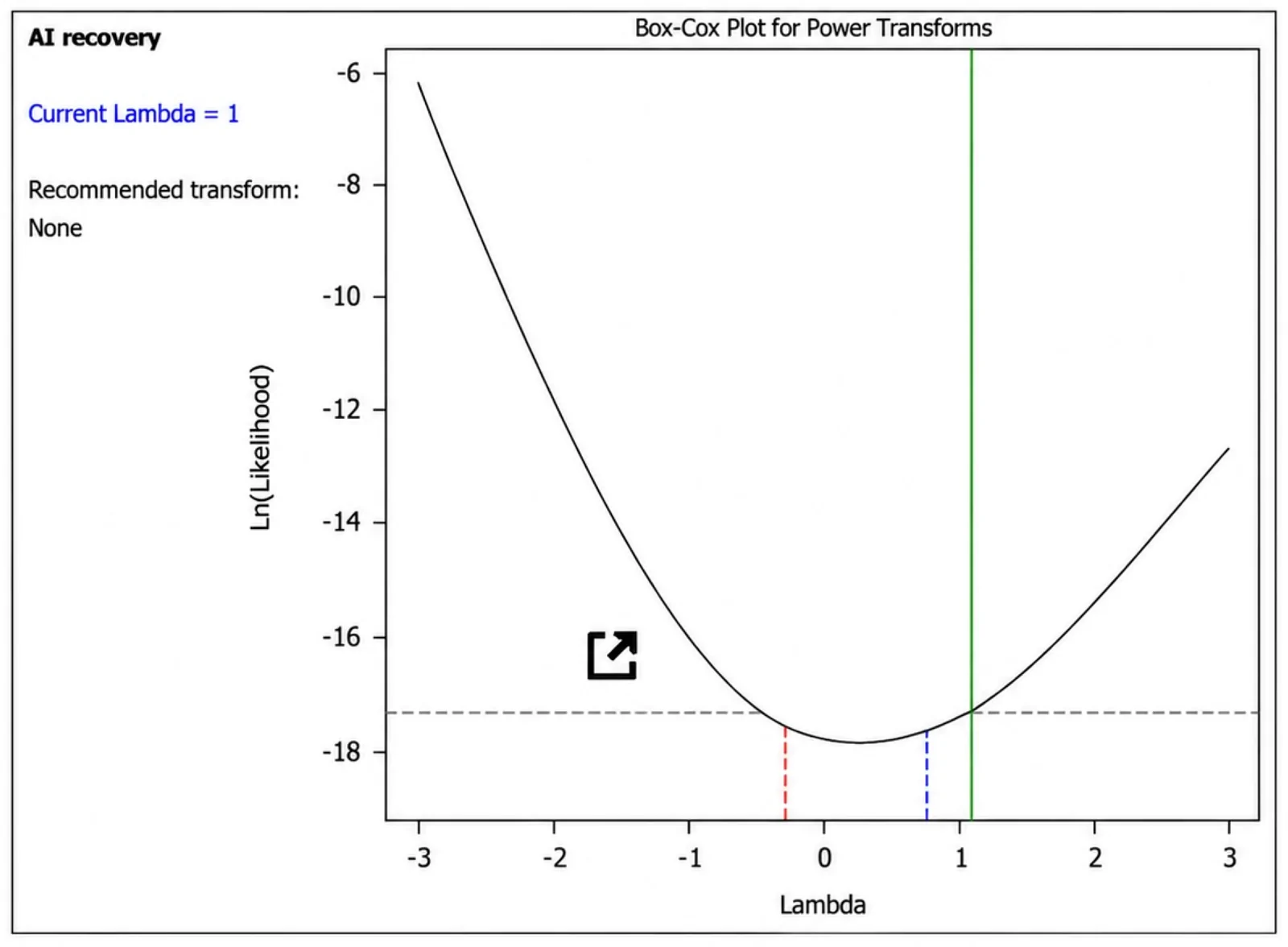
Box–Cox plot for the reduced cubic model of DD Al recovery.

Figure 4 shows a Box–Cox plot for power transforms. This chart identifies the most suitable power transformation for response data. The 95% confidence interval around the optimal lambda value (the curve’s minimum) does not exclude any particular transformation recommended for the reduced cubic model.

### 3.4. Effects of Process Variables on Al Recovery

**Figure 5:**
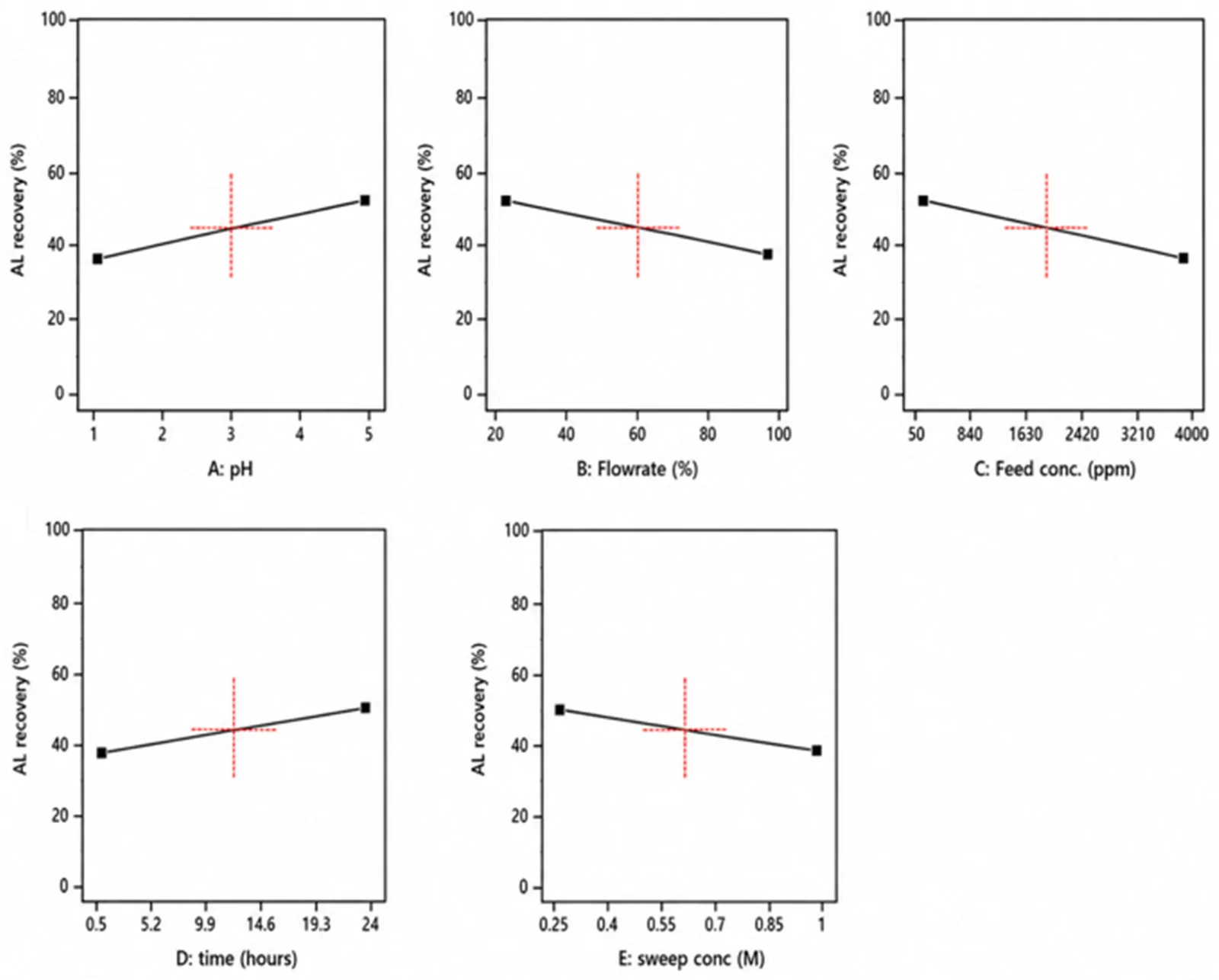
Effects of process variables on Al recovery. (**A**) pH (**B**) flowrate (**C**) feed concentration (**D**) Time (**E**) sweep concentration.

Figure 5 shows the effects of the various process variables on Al recovery. Figure 5A shows the variation in feed pH with Al recovery. The linear relationship indicates that Al recovery increases with increasing pH. This is due to fewer H^+^ ions competing with Al^3+^ ions for exchange sites on the membrane in a low-acidic medium, compared to a high-acidic medium. Also, the effect of feed pH on aluminium recovery is closely related to aluminium speciation. Under strongly acidic conditions, aluminium exists predominantly as Al^3+^, which readily participates in cation exchange across the Nafion membrane. As pH increases, hydrolysis reactions progressively form cationic hydrolysis species such as AlOH^2+^ and Al(OH)_2_^+^, which remain transportable through the cation-exchange membrane. However, further increases in pH favour the precipitation of amorphous Al(OH)_3_ and, at still higher pH values, the formation of anionic Al(OH)_4_^−^ species. These changes reduce the concentration of mobile cationic aluminium species available for Donnan dialysis. Consequently, an optimum pH exists at which sufficient dissolved cationic aluminium species are available while excessive hydrolysis and precipitation are avoided. Figure 5B shows the relationship between Al recovery and feed flow rate. The linear relationship indicates that Al recovery decreases with increasing feed flow rate. This is due to reduced contact time between the solution and the membrane, minimising ion exchange at higher feed flow rates. Lowering the feed flow rate maximises the time the solutions remain on the membrane, providing more time for Al^3+^ ions to diffuse through it. Figure 5C shows the effects of feed concentration on Al recovery. The linear relationship indicates that Al recovery decreases with increasing concentration. This is due to membrane site saturation at a higher feed concentration, resulting in fewer Al^3+^ ions being transported. Aluminium transport across Nafion 117 is governed by the combined effects of the Donnan potential, concentration gradients, membrane permselectivity, and competitive ion exchange. Because Nafion is a cation-exchange membrane containing fixed negatively charged sulfonate (–SO_3_^−^) functional groups, cation transport occurs through the exchange of aluminium species in the feed solution with protons originating from the sweep solution, while co-ions are excluded by the Donnan equilibrium. Consequently, the transport rate depends not only on the aluminium concentration gradient but also on proton activity, membrane permselectivity, and the ionic strength of the surrounding electrolyte [22]. Figure 5B shows the variation in runtime with Al recovery. The linear relationship indicates that Al recovery increases with increasing runtime. This is because the longer the runtime, the more time the ions have to be transported or the more time there is to achieve equilibrium, compared to a shorter runtime.

Figure 5E shows the relationship between Al recovery and sweep concentration. The linear relationship indicates that Al recovery decreases with increasing sweep concentration. This is due to increased osmotic dehydration of the membrane structure as the sweep concentration approaches 1M, leading to reverse ion transport, reduced Donnan exclusion, and increased water transport [23]. In Donnan dialysis, ion movement primarily depends on the electrochemical potential (Donnan potential) generated by the concentration difference of counter-ions across the cation-exchange membrane, rather than on hydraulic pressure [24]. Raising the sweep acid concentration initially boosts the proton gradient, encouraging H^+^ ions in the sweep compartment to exchange with aluminium cations in the feed solution [23]. However, very high sweep concentrations can raise the ionic strength of the receiving solution, potentially compressing the electrical double layer within the membrane, decreasing the effective Donnan potential, and increasing competition between protons and aluminium species for the negatively charged sulfonic acid exchange sites [25]. As a result, the overall aluminium flux might decline despite the increased acid concentration gradient. Figure 4 depicts one-factor-at-a-time main-effect trends, with all other variables held constant at their median values. Since the final reduced cubic model includes significant interaction and higher-order terms (see Table 4 and Table 5), these marginal trends alone do not fully capture the response of aluminium recovery. Consequently, the combined effects of the operating variables are further analysed through interaction analysis in Section 3.5.

### 3.5. Factor Interaction Plots for the Reduced Cubic DD Al Recovery Model

**Figure 6:**
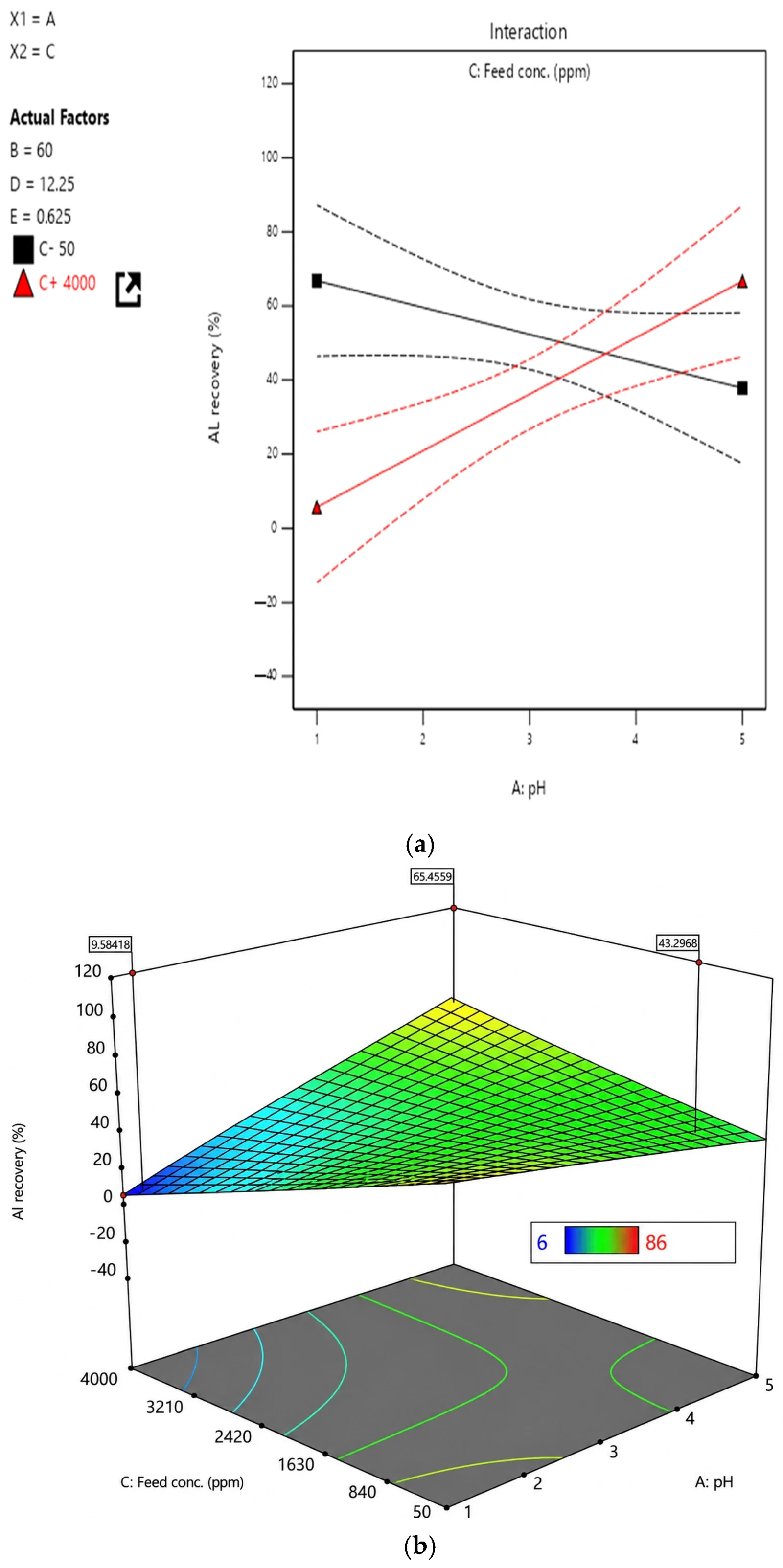
pH and feed concentration interaction influence on Al recovery: (**a**) line plot; (**b**) 3D plot.

WTPS is a byproduct generated during the treatment of drinking water through alum coagulation, primarily consisting of Al (OH)_3_ precipitate [22], which was used in this study, compared to Al_2_(SO_4_)_3_ used by Asante-Sackey et al. [26], which is not a major component of WTPS. The Al_2_(SO_4_)_3_ generates highly soluble Al^3+^ ions with enhanced electrostatic driving forces [27]. This amplifies the importance of individual process variables in RSM analysis. In contrast, in Al (OH)_3_ solution, aluminium predominantly occurs as hydrolysed and polymeric species with restricted mobility [28]. This will influence the two-factor interaction effect on Al recovery. Consequently, efficient aluminium transport also transpires when two factors operate concurrently. Consequently, substantial two-factor interaction effects predominate the response variable. This is evident in the ANOVA analysis in Table 4, where the two-factor interaction variables have a highly significant effect on Al recovery (*p* < 0.005). Hence, the focus is on the two-factor interactions. Figure 6a shows predicted Al recovery versus pH/feed concentration plots. The other parameters have been considered at mean values (feed flow rate: 60%, runtime: 12.25 h, sweep concentration: 0.625 M). The straight lines in the graph cross, which indicates a significant influence on Al recovery by the interaction between feed pH and feed ((Al (OH)_3_) concentration. This indicates that aluminium recovery cannot be predicted without concurrent knowledge of the feed pH and feed concentration. Also, to maximise Al recovery, the model indicates that a high pH (5 maximum) is required while maintaining a high feed concentration, as shown in Figure 6b.

**Figure 7:**
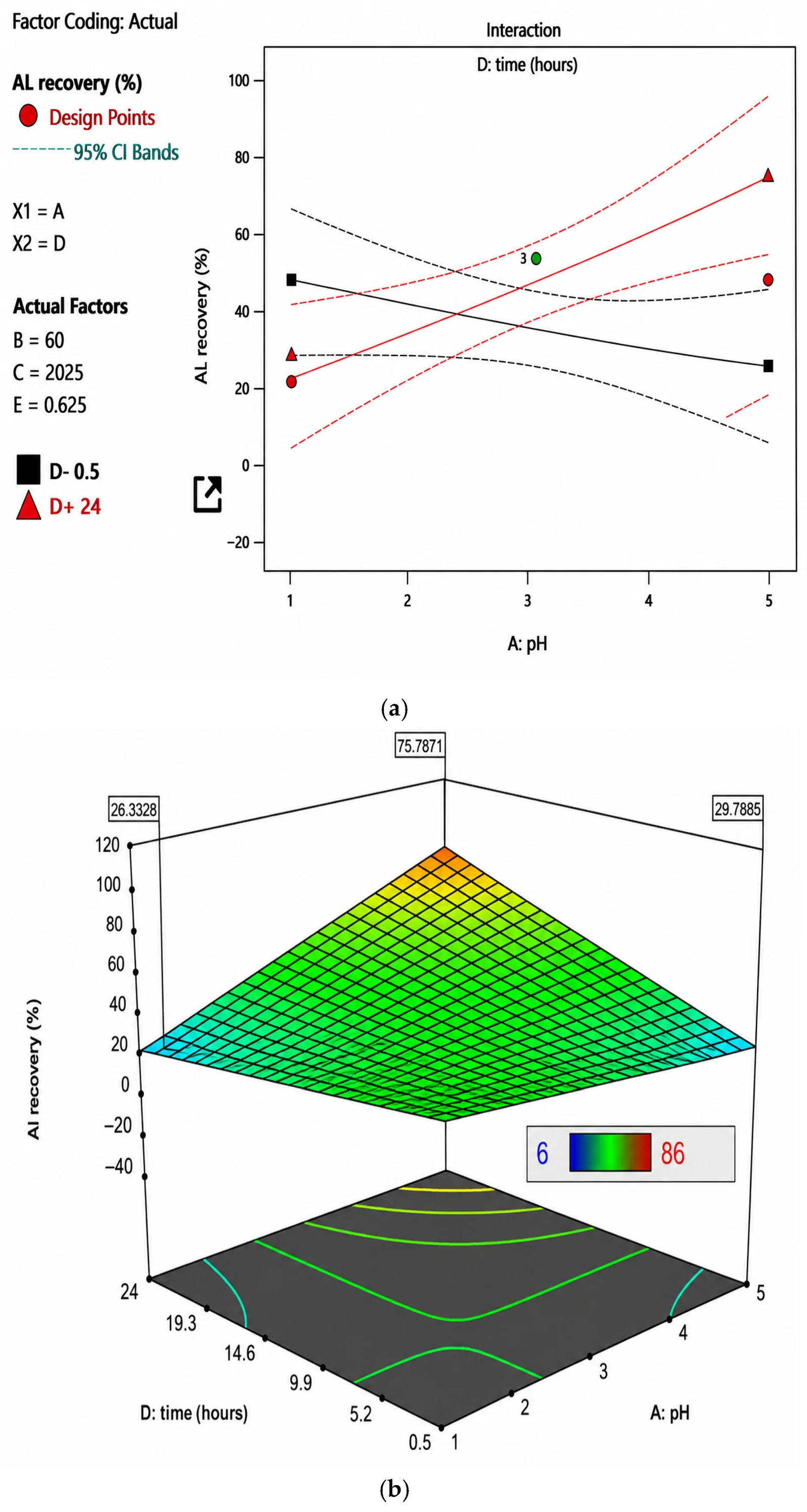
pH and time interaction influence on Al recovery: (**a**) line plot; (**b**) 3D plot.

Figure 7a shows predicted Al recovery versus pH/time interaction plots. The other parameters have been considered at mean values (feed flow rate: 60%, feed concentration: 2025 ppm, sweep concentration: 0.625 M). The straight lines in the graph cross, indicating a significant interaction between feed pH and time on Al recovery. This indicates that aluminium recovery cannot be predicted without concurrent knowledge of the feed pH and time. It can be observed that increases in pH and process time simultaneously increased Al recovery, as shown in Figure 7b. This can also be confirmed from Table 5.

**Figure 8:**
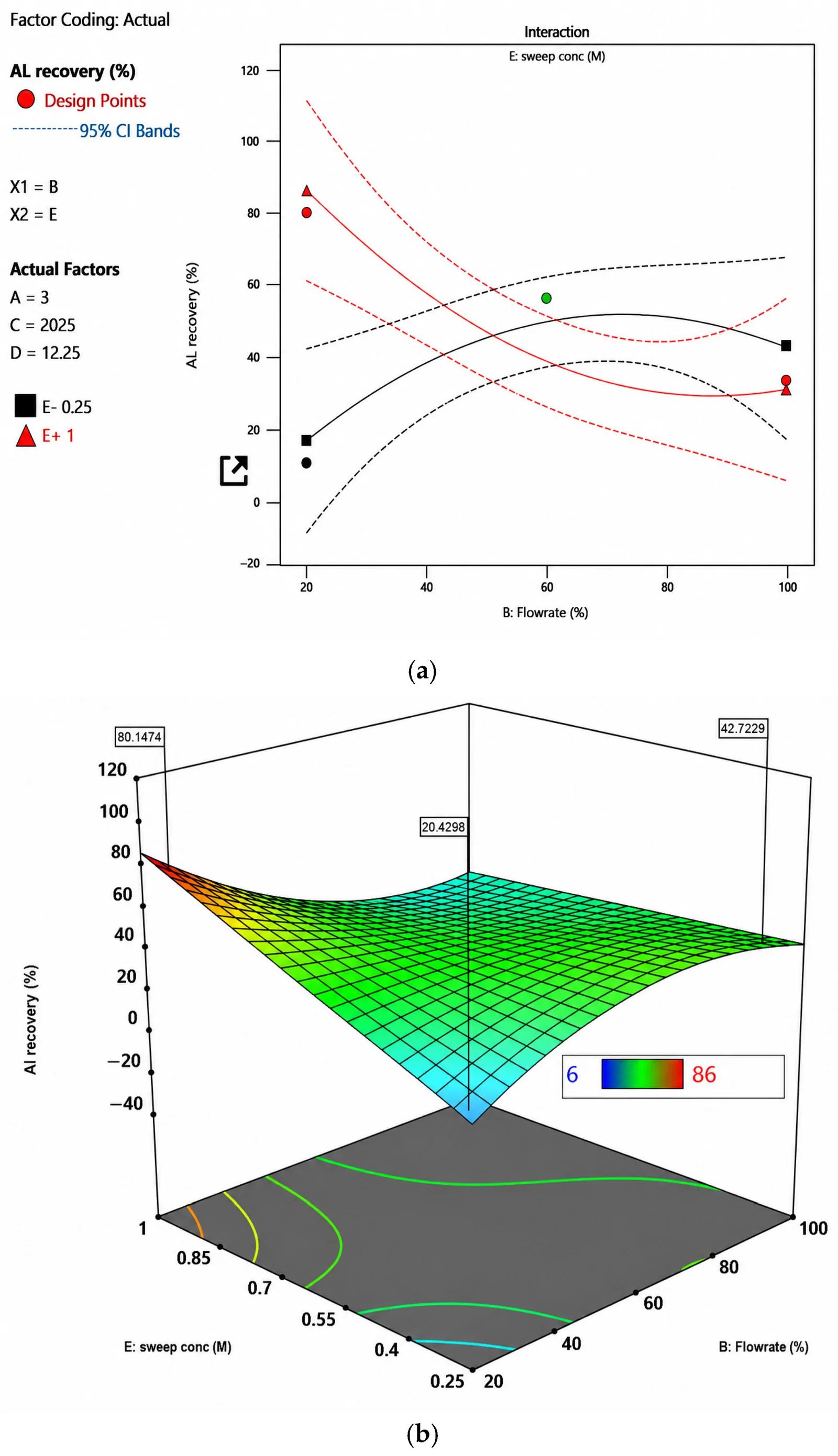
Feed flow rate and sweep concentration interaction influence on Al recovery: (**a**) line plot; (**b**) 3D plot.

Figure 8a shows predicted Al recovery versus feed flow rate/sweep concentration interaction plots. The other parameters have been set to their mean values (Feed pH: 3, Feed concentration: 2025 ppm, time: 12.25 h). It can be observed that there is an interaction between the feed flow rate and sweep concentration for Al recovery. The results of ANOVA also show that the *p*-value of the interaction coefficient between feed flow rate and sweep concentration is less than 0.005 for Al recovery, indicating its significance. In contrast, increasing the flow rate at high concentrations decreases Al recovery, as shown in Figure 8b. This is due to reduced contact time between ions and the membrane surface, hindering effective penetration.

**Figure 9:**
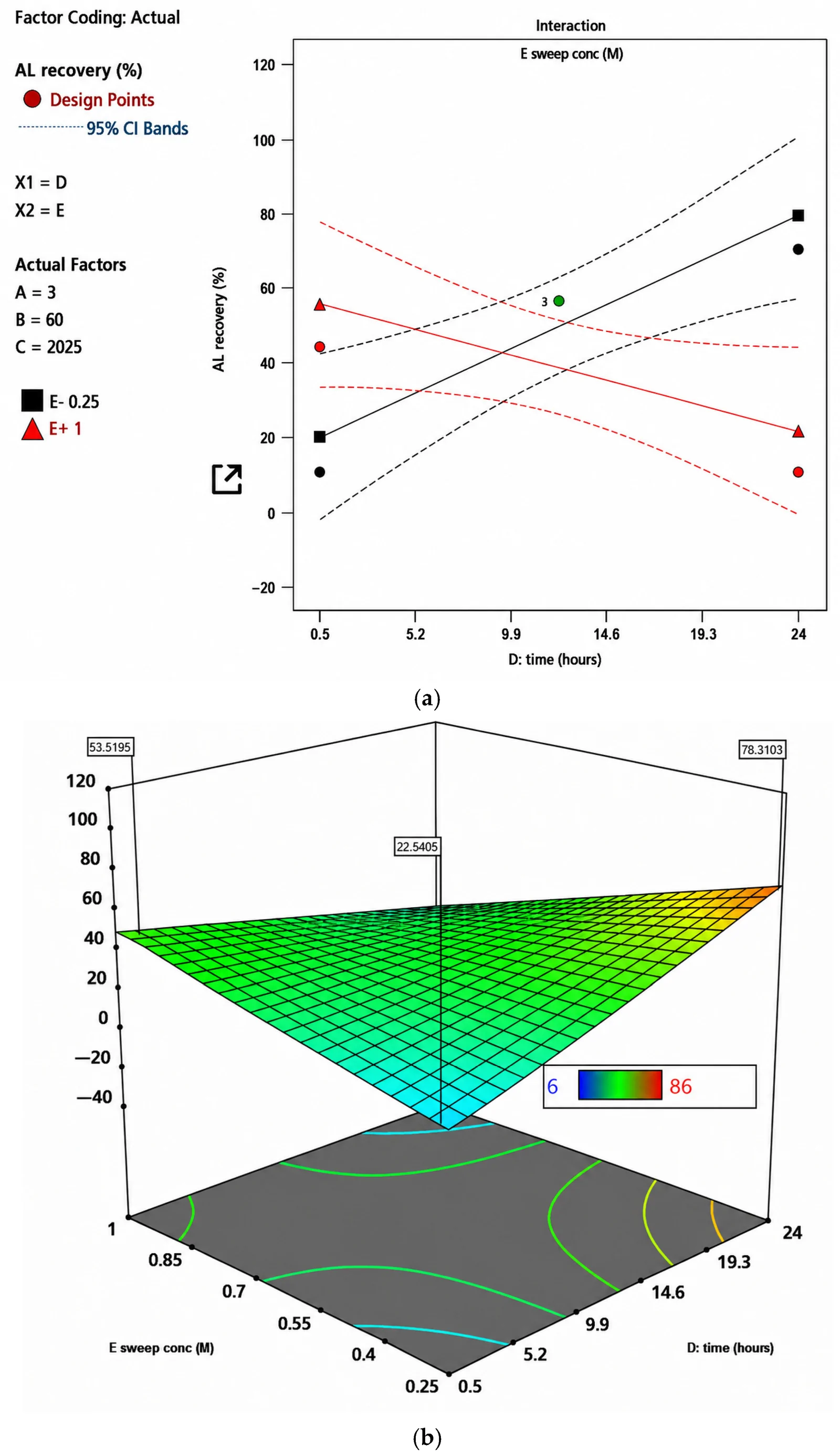
Time and sweep concentration interaction influence on Al recovery: (**a**) line plot; (**b**) 3D plot.

Figure 9a,b illustrates the predicted aluminium (Al) recovery as a function of time and sweep concentration interactions. Other experimental parameters were held constant at their mean values: feed pH at 3, feed flow rate at 60%, feed concentration at 2025 ppm, and sweep concentration at 0.625 M. The observed interaction between time and sweep concentration suggests that the dynamics of Al recovery are significantly influenced by the sweep agent’s concentration over the course of the process, suggesting potential optimisation opportunities. Additionally, the ANOVA results (Table 4 and Table 5) indicate that the interaction coefficient between feed concentration and sweep concentration is statistically significant (*p* < 0.005), underscoring the combined effect of these variables on Al recovery. This underscores the importance of carefully controlling both feed and sweep concentrations to maximise recovery efficiency and highlights the complex interplay of operational parameters that influence adsorption and desorption mechanisms in the process.

### 3.6. Optimisation of DD Al Recovery

Optimal conditions were meticulously determined to maximise aluminium (Al) recovery in the process. The study employed the desirability function-based response surface methodology (DD-RSM) in Design-Expert 13, alongside Particle Swarm Optimisation (PSO) implemented in Python 3.13. Table 6 provides a comprehensive overview of the independent variables and their respective response limits considered during the optimisation process, highlighting the multidimensional approach used to enhance Al recovery.

Table S3 shows predicted optimal conditions for the goals from BBD-RSM. Although several optimisation solutions generated by the reduced cubic response surface exhibited desirability values of 1.0, some predicted aluminium recoveries exceeded the physically attainable limit of 100%. These values are not physically meaningful; rather, they reflect the unconstrained nature of polynomial response surface models, which may extrapolate beyond the experimental design space during mathematical optimisation. Consequently, only physically feasible optimisation solutions were considered for practical evaluation. The occurrence of predicted recoveries exceeding 100% reflects a limitation of unconstrained polynomial response surface models rather than the Donnan dialysis process itself. Response surface methodology employs empirical polynomial equations to approximate the true response surface within the experimental design space; consequently, mathematical optimisation may yield extrapolated optima outside the physically meaningful response range when no response constraints are imposed [29,30,31]. Therefore, predictions above 100% should be interpreted as artefacts of the regression model rather than achievable process performance. To obtain a realistic optimum, Particle Swarm Optimisation (PSO) was subsequently employed because it searches within the defined design space while avoiding unrealistic polynomial extrapolation.

**Figure 10:**
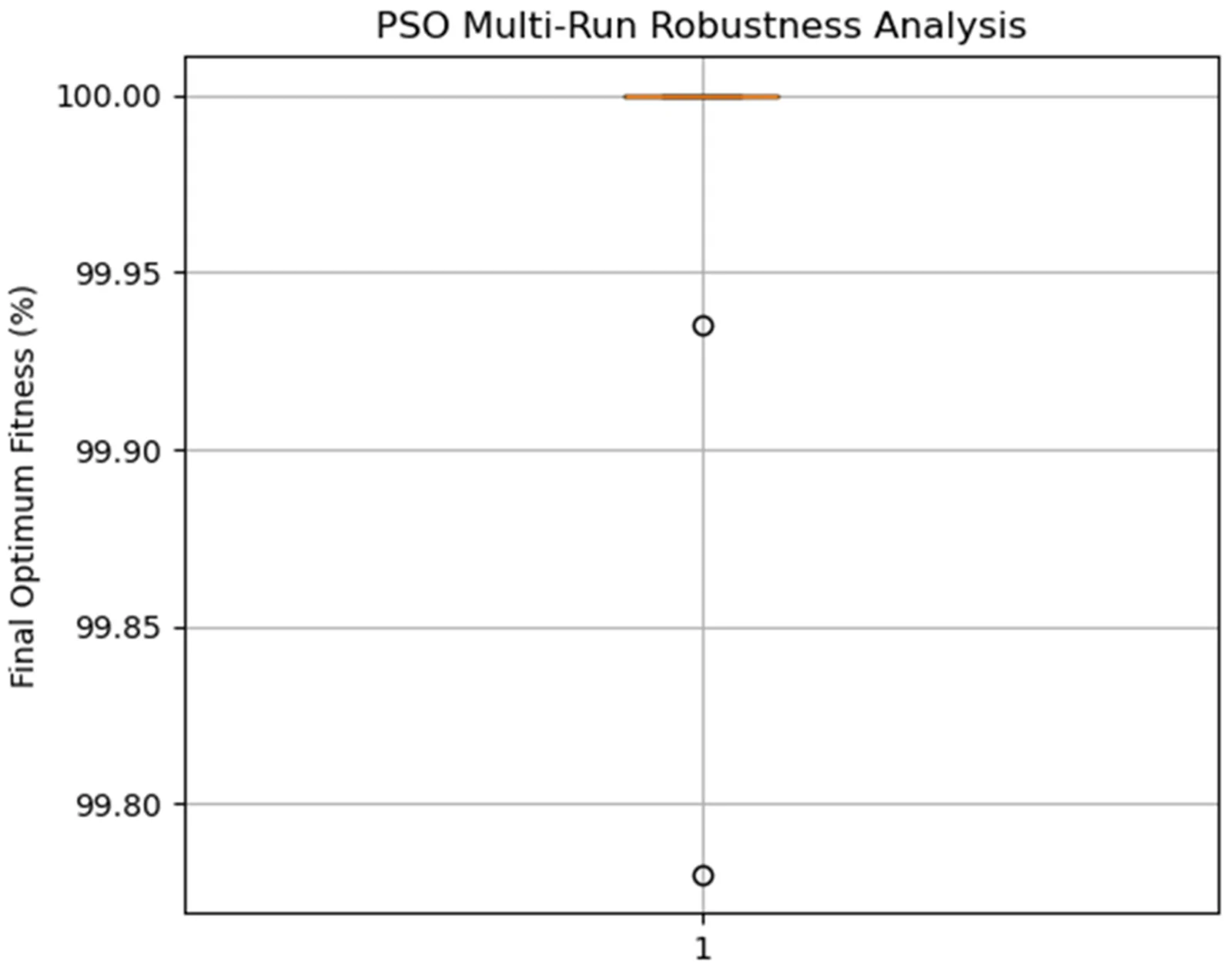
PSO multi-run robustness analysis for DD Al recovery optimisation.

Based on the results in Table S3, the optimal conditions for maximum DD Al recovery are a pH of 4.97, a feed flow rate of 23%, a feed concentration of 2823.5 ppm, a time of 8.9 h, and a sweep concentration of 0.97 M. Given the limitations of BBD-RSM, PSO in Python software was used to optimise the DD Al recovery process, using the optimal conditions in Table 6, the experimental design, and the Al recovery in Table S1. According to PSO, the optimal conditions were a pH of 4.74, a feed flow rate of 99%, a feed concentration of 1313.30 ppm, a time of 21.5 h, and a sweep concentration of 0.25 M, yielding a maximised Al recovery of 99.1%. The notable differences between the operating conditions predicted by RSM and PSO stem from their different optimisation strategies. RSM’s numerical optimisation, based on the desirability function approach by Derringer and Suich [32], performs local optimisation of the response surface and can converge to different stationary points depending on initial conditions and the model’s complexity, especially for higher-order polynomials. Conversely, Particle Swarm Optimisation (PSO) is a global, population-based algorithm that explores multiple regions simultaneously through cooperative sharing among particles, lowering the risk of settling in local optima [33,34]. Since PSO extensively searches the entire feasible design space, it can find alternative conditions that may lead to a better global optimum. In this study, the PSO optimal solution aligned more closely with experimental results than the RSM solution, indicating that PSO identified a more practically achievable operating point rather than just fitting mathematical artefacts of the model. Figure 10 plots the independent PSO optimisation iterations against the maximum recovery of 99.1%, indicating 100% relative algorithmic fitness. The narrow interquartile range at this maximum confirms that PSO consistently converges to the true global maximum. This demonstrates that PSO effectively resolved and bypassed the overfitting issues faced by BBD-RSM. To confirm the model’s accuracy, the optimal conditions were applied in the experimental test. Table 7 compares the experimental and modelling optimisation results. The experimental data align with the conditions predicted by PSO, whereas the BBD-RSM prediction does not match the experimental results. This highlights the limitations of RSM optimisation for complex processes when using cubic models. The 9.2% deviation between predicted and experimental recovery for PSO is considered acceptable for Donnan dialysis systems, which are governed by coupled transport, concentration polarisation, and pH-dependent speciation effects. Similar deviation has been widely reported in a membrane-based and adsorption-related optimisation study by Peydayesh et al. [35]. Experimental uncertainty may arise from instrument limitations in atomic absorption spectroscopy (AAS), minor fluctuations in feed composition, and inherent variability in membrane transport processes. These factors collectively contribute to deviations between predicted and observed responses. Unlike previous Donnan dialysis optimisation studies that investigated relatively simple soluble feed solutions [7,21], this study considers a hydroxide-rich synthetic water treatment sludge, where aluminium transport is governed by pH-dependent hydrolysis and dissolution equilibria. These characteristics introduce stronger nonlinear interactions among process variables, as evidenced by the reduced cubic response surface model. While RSM adequately described the process behaviour, it produced several unrealistic optimisation solutions, with predicted recoveries exceeding 100%, highlighting its limitations in highly nonlinear systems. The incorporation of PSO enabled efficient exploration of the response surface and identified a feasible global optimum that closely matched the experimental validation. This demonstrates the suitability of hybrid optimisation for complex Donnan dialysis applications involving realistic sludge-derived feed streams.

## 4. Conclusions

This study effectively modelled and optimised aluminium recovery from an acidified synthetic aluminium hydroxide solution that mimics water treatment plant sludge using the Donnan dialysis process. A Box–Behnken design combined with response surface methodology (BBD–RSM) was used to assess the effects of feed pH, feed flow rate, feed concentration, runtime, and sweep concentration on aluminium recovery. Results indicated that feed concentration had the strongest negative effect, while runtime and sweep concentration showed significant positive interaction effects. The importance of these interactions underscores the complex transport behaviour of aluminium species from aluminium hydroxide, in contrast to more common aluminium sulphate systems. The initial 2FI model had limited predictive accuracy (R^2^ = 0.5503), but the reduced cubic model improved fit and captured nonlinear interactions. Nonetheless, optimisation using this model sometimes predicted recovery rates over 100%, revealing the limitations of polynomial-based methods for highly nonlinear Donnan dialysis systems. To address this, Particle Swarm Optimisation (PSO) was integrated with the model, successfully identifying the optimal operating conditions. The PSO predicted a maximum aluminium recovery of 99.1% at pH 4.74, feed flow rate of 98.6%, feed concentration of 1313.30 ppm, runtime of 21.51 h, and sweep solution concentration of 0.25 M. Under these conditions, experimental validation achieved a 90.4% recovery, with a prediction error of only 9.2%. In contrast, the RSM-optimised result had a much higher error of 67%. These results demonstrate the superior robustness and accuracy of PSO for optimising complex Donnan dialysis systems. Overall, this study offers new insights into the transport behaviour of aluminium hydroxide-rich feeds, develops predictive models linking operational variables to recovery, and highlights the benefits of combining RSM with metaheuristic optimisation. This study advances previous research on Donnan dialysis optimisation by investigating aluminium recovery from a hydroxide-rich synthetic water treatment sludge that more closely reflects the composition of practical sludge than the soluble aluminium salt systems commonly reported. The results demonstrate that the complex nonlinear behaviour of aluminium hydroxide limits the effectiveness of conventional RSM optimisation, whereas PSO successfully identified experimentally achievable global optimum conditions, with substantially improved agreement between predicted and measured recoveries. The results indicate that integrating RSM with PSO is a promising strategy for optimising Donnan dialysis operating parameters and improving aluminium recovery from hydroxide-rich model systems. However, the findings should be interpreted within the limitations of this study. Future research should enhance the validation of modelling results by integrating hybrid optimisation techniques. Specifically, combining artificial neural networks (ANNs) is recommended to boost prediction accuracy and improve model efficiency. This hybrid approach can more effectively model complex nonlinear relationships in the Donnan dialysis process and yield more robust and dependable optimisation results than using a single method.

## Figures and Tables

**Table 1:** Experimental design conditions and factor levels using a BBD adapted from RSM.

Process	Input Variables		Coded Levels (X)		
**DD Aluminium Ions Recovery Process**			−1	0	+1
	X_1_	pH of Feed Solution	1	3	5
	X_2_	Feed Flow Rate (%)	20	60	100
	X_3_	Feed Concentration (mg/L)	50	2025	4000
	X_4_	Runtime (h)	0.5	12.25	24
	X_5_	Sweep Concentration (M)	0.25	0.625	1.0
**Parameter**			**Description**		
Design			Box–Behnken Design (BBD)		
Number of Factors			5		
Levels			3		
Total Experimental Runs			43		
Factorial Runs			40		
Centre Points Replication			3		
Randomisation			Randomised using BBD		

**Table 2:** ANOVA of 2FI model obtained.

Source	Sum of Squares	df	Mean Square	F-Value	*p*-Value	
**Model**	11,955.00	15	797.00	2.20	0.0359	significant
A-pH	1024.00	1	1024.00	3.14	0.0863	
B-Flowrate	45.56	1	45.56	0.1399	0.7110	
C-Feed conc.	1040.06	1	1040.06	3.19	0.0840	
D-time	612.56	1	612.56	1.88	0.1804	
E-sweep conc.	315.06	1	315.06	0.9675	0.3332	
AB	90.25	1	90.25	0.2772	0.6024	
AC	2025.00	1	2025.00	6.22	0.0184	
AD	1332.25	1	1332.25	4.09	0.0656	
AE	81.00	1	81.00	0.2487	0.6216	
BC	121.00	1	121.00	0.3716	0.5467	
BD	64.00	1	64.00	0.1965	0.6607	
BE	1600.00	1	1600.00	4.91	0.0344	
CD	676.00	1	676.00	2.08	0.1600	
CE	812.25	1	812.25	2.49	0.1247	
DE	2116.00	1	2116.00	6.50	0.0162	
**Residual**	9768.91	27	361.81			
Lack of Fit	9768.08	22	444.00	2344.34	<0.0001	significant
Pure Error	0.8333	5	0.1667			
**Cor Total**	21,723.91	42				

**Table 3:** Fit statistics for the 2FI model obtained.

Std. Dev.	19.02	R^2^	0.5503
Mean	45.04	Adjusted R^2^	0.2983
C.V.%	42.23	Predicted R^2^	−0.1705
		Adeq. Precision	6.1192

**Table 4:** ANOVA of reduced cubic model obtained.

Source	Sum of Squares	df	Mean Square	F-Value	*p*-Value	
**Model**	11,135.40	12	927.95	65.43	<0.0001	significant
A: pH	1024.00	1	1024.00	72.21	<0.0001	
B: Flow rate	45.56	1	45.56	3.21	0.0832	
C: Feed conc.	1040.06	1	1040.06	73.34	<0.0001	
D: Time	612.32	1	612.32	43.18	<0.0001	
E: Sweep conc.	5.02	1	5.02	0.3543	0.5562	
AC	2025.00	1	2025.00	142.79	<0.0001	
AD	1332.25	1	1332.25	93.94	<0.0001	
BE	1600.00	1	1600.00	112.82	<0.0001	
CE	812.25	1	812.25	57.28	<0.0001	
DE	2116.00	1	2116.00	149.21	<0.0001	
B^2^E	141.81	1	141.81	10.00	0.0036	
C^2^E	141.81	1	141.81	10.00	0.0036	
**Residual**	425.44	30	14.18			
Lack of Fit	425.44	28	15.19			
Pure Error	0.0000	2	0.0000			
**Cor. Total**	11,560.85	42				

**Table 5:** Fit statistics for reduced cubic model obtained.

Std. Dev.	3.77	R^2^	0.9632
Mean	44.26	Adjusted R^2^	0.9485
C.V.%	8.51	Predicted R^2^	0.9072
		Adeq. Precision	34.2888

**Table 6:** Specified criteria for optimisation.

Name	Goal	Lower Limit	Upper Limit	Lower Weight	Upper Weight	Importance
A: pH	is in range	1	5	1	1	3
B: Flow rate	is in range	20	100	1	1	3
C: Feed conc.	is in range	50	4000	1	1	3
D: Time	is in range	0.5	24	1	1	3
E: Sweep conc.	is in range	0.25	1	1	1	3
AL recovery	maximize	86	90	1	1	3

**Table 7:** The comparison between experimental and modelling optimisation results.

	pH		Feed Flow Rate (%)		Feed Concentration (ppm)		Time (Hours)		Sweep Concentration (M)		Al Recovery (%)	
	BBD-RSM	PSO	BBD-RSM	PSO	BBD-RSM	PSO	BBD-RSM	PSO	BBD-RSM	PSO	BBD-RSM	PSO
Optimisation	4.97	4.47	23	98.6	2823.5	1313.30	8.7	21.5	0.97	0.25	96.98	99.1
Experimental	4.97	4.47	23	98.6	2823.5	1313.30	8.7	21.5	0.97	0.25	32	90.4
Error (%)											67	9.2%

## Data Availability

All datasets supporting the conclusions of this study are included within the manuscript. Additional details can be made available by the corresponding author upon reasonable request.

## Supplementary Materials

The following supporting information can be downloaded at https://www.mdpi.com/article/10.3390/membranes16080256/s1, Table S1: Experimental design using RSM-BBD and Al recovery. Table S2: Mass balance on the DD Al recovery process. Table S3: Predicted optimal conditions.
