# Supplementary material for: Hybrid Response Surface–Particle Swarm Optimisation of Donnan Dialysis Processes for Aluminium Recovery from Water Treatment Sludge
Source: Membranes (Basel). 2026 Jul 27;16(8):256. doi: 10.3390/membranes16080256 (PMC13515296; doi:10.3390/membranes16080256)
Supplement: Supplementary file 1 [file membranes-16-00256-s001.zip › membranes-4385496-supplementary.pdf]

Hybrid Response Surface–Particle Swarm Optimisation of Donnan Dialysis Processes for Aluminium Recovery from Water Treatment Sludge

Supplementary Materials

Table S1. Experimental design using RSM- BBD and Al Recovery.

		**Factor 1**	**Factor 2**	**Factor 3**	**Factor 4**	**Factor 5**	**Response 1**
Std	Run	A:pH	B:Flowrate	C:Feed conc.	D:time	E:sweep conc	AL recovery
			%	ppm	hours	M	%
16	1	5	60	4000	12.25	0.625	86
21	2	3	20	50	12.25	0.625	50
22	3	3	100	50	12.25	0.625	33
28	4	5	60	2025	24	0.625	50
11	5	3	20	2025	12.25	1	80
13	6	1	60	50	12.25	0.625	50
19	7	3	60	2025	0.5	1	44
7	8	3	60	50	24	0.625	83
40	9	3	100	2025	24	0.625	56
38	10	3	100	2025	0.5	0.625	50
34	11	5	60	2025	12.25	0.25	80
6	12	3	60	4000	0.5	0.625	11
12	13	3	100	2025	12.25	1	33
32	14	3	60	4000	12.25	1	63
9	15	3	20	2025	12.25	0.25	11
8	16	3	60	4000	24	0.625	63
25	17	1	60	2025	0.5	0.625	60
27	18	1	60	2025	24	0.625	20
42	19	3	60	2025	12.25	0.625	56
4	20	5	100	2025	12.25	0.625	50
14	21	5	60	50	12.25	0.625	33
39	22	3	20	2025	24	0.625	33
36	23	5	60	2025	12.25	1	62
26	24	5	60	2025	0.5	0.625	17
41	25	3	60	2025	12.25	0.625	56
30	26	3	60	4000	12.25	0.25	6
10	27	3	100	2025	12.25	0.25	44
3	28	1	100	2025	12.25	0.625	33
23	29	3	20	4000	12.25	0.625	50
2	30	5	20	2025	12.25	0.625	50
5	31	3	60	50	0.5	0.625	83
17	32	3	60	2025	0.5	0.25	11
20	33	3	60	2025	24	1	11
29	34	3	60	50	12.25	0.25	50
31	35	3	60	50	12.25	1	50
37	36	3	20	2025	0.5	0.625	11
18	37	3	60	2025	24	0.25	70

15	38	1	60	4000	12.25	0.625	13
24	39	3	100	4000	12.25	0.625	11
1	40	1	20	2025	12.25	0.625	52
35	41	1	60	2025	12.25	1	36
43	42	3	60	2025	12.25	0.625	56
33	43	1	60	2025	12.25	0.25	36

Table S2. Mass Balance on the DD Al Recovery Process.

**RUN**	**INITIAL FEED DISSOLVED Al CONC. (F_1) (mg/L)**	**FINAL FEED DISSOLVED Al CONC. (F_2) (mg/L)**	**FINAL SWEEP DISSOLVED Al CONC. (S_2) (mg/L)**	**SUM EXITING DISSOLVED Al CONC. ($F_2 + S_2$) (mg/L)**	**MASS BALANCE GAP ($F_1 - SUM$)(mg/L)**	**CALCULATED Al LOSS (%)**
1	809.1	359.6	449.5	809.1	0.0	0.0%
2	6023.3	5663.7	359.6	6023.3	0.0	0.0%
3	809.1	359.6	449.5	809.1	0.0	0.0%
4	1078.8	719.2	359.6	1078.8	0.0	0.0%
5	539.4	89.9	449.5	539.4	0.0	0.0%
6	809.1	359.6	440.5	800.1	9.0	1.1%
7	809.1	449.5	359.6	809.1	0.0	0.0%
8	269.7	179.8	89.9	269.7	0.0	0.0%
9	269.7	179.8	89.9	269.7	0.0	0.0%
10	899	269.7	629.3	899.0	0.0	0.0%
11	809.1	719.2	89.9	809.1	0.0	0.0%
12	719.2	269.7	449.5	719.2	0.0	0.0%
13	809.1	719.2	89.9	809.1	0.0	0.0%
14	809.1	719.2	89.9	809.1	0.0	0.0%
15	179.8	89.9	89.9	179.8	0.0	0.0%
16	179.8	89.9	89.9	179.8	0.0	0.0%
17	809.1	719.2	89.9	809.1	0.0	0.0%
18	449.5	89.9	359.6	449.5	0.0	0.0%
19	449.5	89.9	359.6	449.5	0.0	0.0%
20	809.1	539.4	269.7	809.1	0.0	0.0%
21	809.1	449.5	359.6	809.1	0.0	0.0%
22	539.4	89.9	449.5	539.4	0.0	0.0%
23	629.3	269.7	359.6	629.3	0.0	0.0%
24	988.9	629.3	359.6	988.9	0.0	0.0%
25	719.2	269.7	449.5	719.2	0.0	0.0%
26	719.2	359.6	359.6	719.2	0.0	0.0%
27	809.1	359.6	449.5	809.1	0.0	0.0%
28	539.4	449.5	89.9	539.4	0.0	0.0%
29	809.1	719.2	89.9	809.1	0.0	0.0%

30	179.8	89.9	89.9	179.8	0.0	0.0%
31	449.5	359.6	89.9	449.5	0.0	0.0%
32	179.8	89.9	89.9	179.8	0.0	0.0%
33	809.1	539.4	269.7	809.1	0.0	0.0%
34	179.8	89.9	89.9	179.8	0.0	0.0%
35	809.1	359.6	449.5	809.1	0.0	0.0%
36	179.8	89.9	89.9	179.8	0.0	0.0%
37	988.9	629.3	359.6	988.9	0.0	0.0%
38	1348.5	539.4	809.1	1348.5	0.0	0.0%
39	809.1	719.2	89.9	809.1	0.0	0.0%
40	1438.4	1258.6	179.8	1438.4	0.0	0.0%
41	809.1	359.6	449.5	809.1	0.0	0.0%
42	1168.7	449.5	719.2	1168.7	0.0	0.0%
43	629.3	89.9	539.4	629.3	0.0	0.0%

Table S3. Predicted optimal conditions.

Number	pH	Flowrate	Feed conc.	time	sweep conc	AL recovery	Desirability	
1	1.080	31.158	429.127	5.411	0.923	93.949	1.000	
2	1.018	62.521	62.540	0.689	0.829	90.700	1.000	
3	3.444	70.730	1746.893	23.265	0.272	90.186	1.000	
4	2.647	84.991	916.741	23.887	0.269	94.954	1.000	
5	4.921	41.598	3834.709	12.897	0.941	99.597	1.000	
6	4.595	43.840	3981.751	17.629	0.961	96.825	1.000	
7	2.770	21.942	3842.767	1.019	0.986	97.132	1.000	
8	3.716	20.233	2377.047	12.043	0.995	91.519	1.000	
9	4.776	82.457	2065.776	22.708	0.408	95.255	1.000	
10	4.939	65.815	1470.367	22.875	0.283	99.842	1.000	
11	2.363	21.951	187.860	8.206	0.900	92.042	1.000	
12	2.641	90.918	528.558	23.175	0.273	92.279	1.000	
13	2.381	78.634	439.499	23.900	0.270	91.741	1.000	
14	2.365	28.985	83.460	3.006	0.949	90.173	1.000	
15	1.065	27.733	69.371	0.685	0.889	101.722	1.000	
16	1.610	99.202	128.041	23.977	0.310	90.504	1.000	
17	3.520	20.632	2538.369	10.673	0.978	90.424	1.000	
18	1.016	49.040	278.509	0.732	0.913	91.524	1.000	
19	2.684	87.278	202.156	23.090	0.260	91.680	1.000	
20	1.444	98.108	149.141	1.219	0.829	92.585	1.000	
**21**	**4.967**	**23.305**	**2823.546**	**8.677**	**0.967**	**96.982**	**1.000**	**Selected**
22	4.658	57.854	1484.831	22.195	0.269	90.503	1.000	
23	3.855	21.665	2765.299	14.917	0.998	92.201	1.000	
24	4.987	68.232	3056.255	20.617	0.251	90.154	1.000	
25	2.169	75.153	72.469	23.972	0.256	90.055	1.000	
26	4.856	84.438	1681.974	22.937	0.356	101.216	1.000	
27	2.629	94.140	1055.900	23.949	0.260	96.838	1.000	
28	1.170	52.398	106.853	0.832	0.997	98.251	1.000	
29	3.188	98.574	3964.017	0.929	0.984	91.866	1.000	
30	4.401	20.152	2035.598	8.965	0.996	90.568	1.000	
31	4.732	20.634	3011.168	10.965	0.850	90.364	1.000	

32	4.814	75.194	2623.548	22.375	0.357	95.574	1.000
33	1.095	34.723	184.551	10.624	0.984	91.332	1.000
34	3.448	20.654	150.290	13.987	0.994	91.119	1.000
35	4.901	44.624	3870.499	14.189	0.886	92.976	1.000
36	2.270	21.599	3783.554	10.509	0.962	90.921	1.000
37	1.848	98.547	233.517	0.740	0.957	94.429	1.000
38	4.921	79.724	3278.481	22.603	0.450	92.984	1.000
39	1.053	21.370	491.516	1.615	0.899	98.726	1.000
40	3.119	24.216	3695.196	7.701	0.960	96.428	1.000
41	4.551	56.147	1912.069	23.262	0.275	91.718	1.000
42	4.955	87.925	898.821	21.912	0.314	94.701	1.000
43	4.745	98.372	2278.145	23.079	0.467	95.349	1.000
44	1.498	93.651	253.262	0.895	0.962	95.089	1.000
45	1.283	93.792	65.478	23.850	0.283	91.075	1.000
46	3.984	66.547	952.348	23.821	0.307	91.728	1.000
47	4.997	42.737	3989.303	10.816	0.858	93.643	1.000
48	4.862	96.230	189.451	23.640	0.396	90.655	1.000
49	3.595	33.586	3842.804	2.207	0.978	91.294	1.000
50	3.711	68.974	998.008	23.023	0.273	91.565	1.000
51	2.728	76.954	981.947	23.291	0.267	90.400	1.000
52	4.989	71.388	3878.064	23.433	0.528	92.782	1.000
53	4.083	99.123	3956.755	0.980	0.929	90.324	1.000
54	1.338	28.758	207.129	10.389	0.932	93.250	1.000
55	4.617	88.761	3628.690	23.591	0.438	92.744	1.000
56	3.600	22.455	3629.095	11.130	0.989	107.248	1.000
57	4.050	93.738	494.421	23.851	0.262	102.810	1.000
58	3.576	21.133	181.533	8.396	0.973	90.160	1.000
59	4.676	96.712	3983.391	23.993	0.866	99.621	1.000
60	4.976	63.116	3970.387	23.805	0.571	90.558	1.000
61	4.929	81.430	1578.485	21.657	0.254	102.618	1.000
62	4.103	85.415	1375.578	23.362	0.416	90.136	1.000
63	4.737	21.630	3462.368	20.373	0.844	93.053	1.000
64	2.478	90.831	490.975	23.250	0.293	90.434	1.000
65	4.996	92.268	3433.059	23.095	0.554	96.616	1.000
66	3.427	20.616	685.961	8.738	0.998	90.064	1.000
67	4.431	65.501	1295.142	23.309	0.334	91.810	1.000
68	1.639	29.735	172.062	7.877	0.918	92.528	1.000
69	4.141	95.666	3919.375	0.727	0.947	90.489	1.000
70	3.868	66.587	2100.210	23.973	0.269	93.467	1.000
71	2.417	82.769	632.476	23.852	0.298	90.656	1.000
72	4.705	87.899	2367.187	23.952	0.505	94.809	1.000
73	3.705	30.028	3957.351	1.494	0.989	98.917	1.000
74	1.876	22.969	136.001	7.687	0.851	91.023	1.000
75	1.552	20.262	208.094	10.339	0.826	90.984	1.000
76	3.849	21.178	2949.070	9.078	0.996	99.542	1.000
77	1.115	50.458	182.664	1.976	0.914	90.044	1.000
78	4.426	76.782	835.185	22.568	0.302	93.846	1.000
79	2.911	21.805	265.074	12.089	0.971	91.423	1.000
80	2.547	97.819	1694.182	23.979	0.261	91.660	1.000
81	4.857	87.268	2827.067	20.835	0.312	91.791	1.000
82	4.928	82.659	2217.271	23.878	0.510	94.998	1.000

83	2.572	23.429	3566.390	9.578	0.979	92.241	1.000
84	2.779	20.260	1145.914	8.404	0.998	90.584	1.000
85	4.808	46.904	3783.346	11.249	0.991	95.553	1.000
86	2.498	20.486	3444.067	6.053	0.993	97.671	1.000
87	2.948	78.396	395.873	23.056	0.261	91.029	1.000
88	4.964	62.092	3943.741	18.052	0.969	90.998	1.000
89	4.955	70.122	2823.934	22.704	0.369	95.951	1.000
90	4.747	20.549	2807.536	15.018	0.978	101.964	1.000
91	4.160	99.341	3892.155	2.304	0.984	91.066	1.000
92	2.529	23.019	3900.759	14.414	0.981	90.550	1.000
93	3.966	28.039	3673.959	12.604	0.919	94.669	1.000
94	1.101	45.953	145.442	1.137	0.890	91.861	1.000
95	2.635	23.274	526.862	9.354	0.972	90.151	1.000
96	4.835	82.601	2970.911	23.696	0.496	95.421	1.000
97	1.076	61.694	55.464	3.298	0.981	90.893	1.000
98	1.728	20.052	1511.405	9.137	0.999	90.252	1.000
99	2.579	96.268	290.760	23.928	0.308	93.024	1.000
100	4.890	20.646	3869.783	12.197	0.890	116.630	1.000
